# Cerebro-Spinal Meningitis

**Published:** 1866-12

**Authors:** 


*Cerebro-Spinal Meningitis.—By S. S. Sewell, M. D., Prof. Of Anatomy in Chicago Medical College.*

We have not had time to bestow upon this work a careful examination. We have looked through it, however, and candor compels us to say, he has dealt ably with the subject. Whilst we can not afford to discard the guess-work of theory, it is very refreshing to deal with facts, and of these the author has given us something substantial.

We anticipate great pleasure and profit from a careful perusal of this little work, and in the mean time commend it to the public as a most meritorious production.

